# A Multidisciplinary Biospecimen Bank of Renal Cell Carcinomas Compatible with Discovery Platforms at Mayo Clinic, Scottsdale, Arizona

**DOI:** 10.1371/journal.pone.0132831

**Published:** 2015-07-16

**Authors:** Thai H. Ho, Rafael Nunez Nateras, Huihuang Yan, Jin G. Park, Sally Jensen, Chad Borges, Jeong Heon Lee, Mia D. Champion, Raoul Tibes, Alan H. Bryce, Estrella M. Carballido, Mark A. Todd, Richard W. Joseph, William W. Wong, Alexander S. Parker, Melissa L. Stanton, Erik P. Castle

**Affiliations:** 1 Division of Hematology and Oncology, Mayo Clinic, Scottsdale, Arizona, United States of America; 2 Department of Urology, Mayo Clinic Hospital, Phoenix, Arizona, United States of America; 3 Division of Biomedical Statistics and Informatics, Mayo Clinic, Rochester, Minnesota, United States of America; 4 Center for Personalized Diagnostics, Biodesign Institute, Arizona State University, Tempe, Arizona, United States of America; 5 Department of Chemistry & Biochemistry, The Biodesign Institute-Center for Personalized Diagnostics, Arizona State University, Tempe, Arizona, United States of America; 6 Department of Biochemistry and Molecular Biology, Mayo Clinic, Rochester, Minnesota, United States of America; 7 Division of Biomedical Statistics and Informatics, Mayo Clinic, Scottsdale, Arizona, United States of America; 8 Division of Anatomic Pathology, Mayo Clinic, Scottsdale, Arizona, United States of America; 9 Division of Hematology and Oncology, Mayo Clinic, Jacksonville, Florida, United States of America; 10 Department of Radiation Oncology, Mayo Clinic, Scottsdale, Arizona, United States of America; 11 Departments of Health Sciences Research, Mayo Clinic, Jacksonville, Florida, United States of America; 12 Department of Laboratory Medicine/Pathology, Mayo Clinic, Scottsdale, Arizona, United States of America; MD Anderson Cancer Center, UNITED STATES

## Abstract

To address the need to study frozen clinical specimens using next-generation RNA, DNA, chromatin immunoprecipitation (ChIP) sequencing and protein analyses, we developed a biobank work flow to prospectively collect biospecimens from patients with renal cell carcinoma (RCC). We describe our standard operating procedures and work flow to annotate pathologic results and clinical outcomes. We report quality control outcomes and nucleic acid yields of our RCC submissions (N=16) to The Cancer Genome Atlas (TCGA) project, as well as newer discovery platforms, by describing mass spectrometry analysis of albumin oxidation in plasma and 6 ChIP sequencing libraries generated from nephrectomy specimens after histone H3 lysine 36 trimethylation (H3K36me3) immunoprecipitation. From June 1, 2010, through January 1, 2013, we enrolled 328 patients with RCC. Our mean (SD) TCGA RNA integrity numbers (RINs) were 8.1 (0.8) for papillary RCC, with a 12.5% overall rate of sample disqualification for RIN <7. Banked plasma had significantly less albumin oxidation (by mass spectrometry analysis) than plasma kept at 25°C (*P*<.001). For ChIP sequencing, the FastQC score for average read quality was at least 30 for 91% to 95% of paired-end reads. In parallel, we analyzed frozen tissue by RNA sequencing; after genome alignment, only 0.2% to 0.4% of total reads failed the default quality check steps of Bowtie2, which was comparable to the disqualification ratio (0.1%) of the 786-O RCC cell line that was prepared under optimal RNA isolation conditions. The overall correlation coefficients for gene expression between Mayo Clinic vs TCGA tissues ranged from 0.75 to 0.82. These data support the generation of high-quality nucleic acids for genomic analyses from banked RCC. Importantly, the protocol does not interfere with routine clinical care. Collections over defined time points during disease treatment further enhance collaborative efforts to integrate genomic information with outcomes.

## Introduction

To support medical research, institutional biobanking efforts have encompassed archived formalin-fixed, paraffin-embedded (FFPE) tissue blocks, frozen tissues, peripheral blood, questionnaires, and medical records [1–4]. Genitourinary diseases are heterogeneous and range from benign to malignant conditions [5,6]. The construction of a biobank encompassing both benign and malignant genitourinary diseases may yield clues regarding molecular progression of disease. In 2010, Mayo Clinic (Scottsdale, Arizona) initiated the Multidisciplinary Genitourinary Diseases Biospecimen Bank to prospectively collect biospecimens from patients with genitourinary diseases and to support health-related research. Herein, we report our experience with frozen banking protocols for renal cell carcinoma (RCC) biospecimens that are compatible with standard clinical practices.

The Cancer Genome Atlas (TCGA) Research Network has analyzed the DNA, RNA, and protein from various human tumors to generate molecular profiles, identify recurrent molecular aberrations, and create public data portals to support medical research [7–11]. Currently, TCGA integrates data from DNA methylation at CpG islands, microarray-based measurement of copy number, whole-exome sequencing, RNA sequencing, and reverse-phase protein arrays. The development of next-generation DNA, RNA, chromatin immunoprecipitation (ChIP) sequencing, and high-throughput protein analyses provides an opportunity for institutions to characterize genomic, transcriptomic, and proteomic alterations. However, the application of established and emerging platforms for molecular analysis depends on well-annotated and high-quality biospecimens. Furthermore, these platforms may necessitate large quantities of frozen biospecimens, requiring standard operating procedures to be in place at the time of tissue collection.

To address an unmet need for banked clinical specimens suitable for high-throughput genomic analysis, we developed a protocol to collect frozen tissue specimens, blood, and urine from patients affected by RCC through longitudinal biospecimen collection during follow-up appointments. We describe our standard operating procedures, work flow, and research coordinator efforts, as well as our quality assessment of biospecimens submitted to TCGA from a single institution. We also assessed the albumin oxidation in banked plasma, a surrogate of plasma quality. It is unknown whether banked specimens collected under routine clinical care are compatible with emerging discovery platforms such as ChIP sequencing. Herein, we demonstrate that our biobanking protocol is compatible with multiple platforms, including DNA, RNA, and ChIP sequencing, as well as mass spectrometry (MS) analysis.

## Materials and Methods

### Patient Eligibility and Recruitment

Patients eligible for enrollment were those seen at Mayo Clinic (Scottsdale, Arizona) who were ≥18 years old, able to provide informed consent, and undergoing evaluation for genitourinary diseases (kidney, urothelial, prostate, testicular, and penile malignancies or benign prostatic hyperplasia or nephrolithiasis). Patients were contacted during routine clinical visits or in preoperative settings within Mayo Clinic departments and divisions, including urology, radiation oncology, pathology, and medical oncology. Patients were excluded if they declined to participate or if the banking of their biospecimens would compromise the availability of tissue for diagnosis and standard clinical care. The protocol for collecting biospecimens was approved by the Mayo Clinic Institutional Review Board. The current informed consent form (S1 File), which was updated in 2012, was approved by the Mayo Clinic Institutional Review Board (protocol no. 08–000980). Patients were enrolled from June 1, 2010, through January 1, 2013. Patients provided written consent and the signed consent form was scanned into the electronic medical record. Patients also received a copy of the signed consent form. This consent procedure was approved by the Mayo Clinic Institutional Review Board.

### Blood and Urine Collection

Blood and urine specimens are collected from patients preoperatively, postoperatively, and during systemic treatments (every 3 months) for routine clinical care. The blood and urine are transferred from the phlebotomist to the laboratory processing area within 30 minutes of collection. Blood is collected in BD Vacutainer tubes (red top, Fisher Scientific 02-685-112, no anticoagulants; lavender top, Fisher Scientific 02-657-32, K_2_ EDTA). Contents of red-top tubes are allowed to clot for 10 to 30 minutes, then centrifuged at 1,882 relative centrifugal force (RCF) for 10 minutes, and serum is transferred into 3×1.8-mL Nunc cryovials (Fisher Scientific 12565167N). Lavender-top tubes are centrifuged at 1,882 RCF in a refrigerated centrifuge; plasma is then divided and placed into 3×1.8-mL Nunc cryovials (Fisher Scientific 12565167N); the remaining buffy coat is separated into 3×1.8-mL Nunc cryovials (Fisher Scientific 12565167N). This protocol yields approximately 4.5 mL of plasma, serum, and buffy coat per collection visit. Samples are stored at −80°C.

For urine collections, 2 mL of urine is divided into 2 cryovials for storage; the remaining urine is centrifuged at 1,882 RCF for 5 minutes in a Hycor KOVA (Fisher Scientific VT87139) tube in a refrigerated centrifuge. The supernatant is removed and the pellet resuspended in 1 mL of urine and stored in a cryovial. Centrifuged samples are stored at −80°C.

### Tissue Collection

Surgical specimens are handled according to standards of clinical care. The specimens are delivered directly from the operating room to the pathology department. After immediate macroscopic review, the pathologist decides whether adequate tissue is available for banking. The time from the start of warm ischemia to freezing can be as short as 10 minutes but as long as 110 minutes. The mean warm-ischemia time (ligation of blood flow) to freezing was 45.8 minutes. The warm-ischemia time is influenced by factors such as partial vs radical nephrectomy or an open vs minimally invasive approach. The tumor sample (and a sample of uninvolved tissue, if available) are macrodissected from the surgical specimen. Each sample is bisected, with 1 FFPE half as a mirror image representing the frozen biobank sample. The mirror-image FFPE sample is used as one of the clinical diagnostic slides. The biobank samples are immediately frozen in 7-mL polycarbonate tubes (Sarstedt 71.9923.610) in a −90°C bath of Novec engineered fluid (3M HFE-7000) cooled in a HistoChill freezing bath (SP Scientific HC80A0). No embedding medium or preservative is added to frozen tissue. Tumor cellularity of the FFPE mirror image is assessed by a pathologist and reported as part of the standard-of-care pathology synoptic report.

### Biospecimen Storage

Unique identification numbers are assigned to each individual container with bar code labels. Biospecimens are stored in a Brooks BioStore −80°C Automated Sample Storage System (Brooks Automation Inc). Samples can be retrieved manually or robotically.

### Biobank Research Electronic Data Capture Database

A coded and secure Research Electronic Data Capture (REDCap) database was constructed with pertinent clinical information and database identifiers. Biospecimens are tracked by unique identification numbers assigned by the Research and Laboratory Information Management System (RLIMS). Pertinent pre-, peri-, and postoperative clinical variables (procedures, complications, and surgical outcomes) are annotated and linked to biospecimen data. In oncologic cases, we also annotate progression, survival, and treatment outcomes from the patient medical record. Clinical follow-up is maintained by data coordinators for matched blood, urine, and tissue (primary nephrectomies, biopsies, and metastectomies).

### TCGA Papillary RCC Sample Submission

For papillary RCC solicited by TCGA (KIRP cases), a genitourinary pathologist (M.L.S.) reviewed the diagnostic slide representing the frozen tumor. Specimens were selected only if they met the standard TCGA biospecimen criteria with a corresponding buffy coat (for germline DNA analysis). The minimum DNA, RNA, and germline DNA yield required was 6.9 μg, 5.15 μg, and 4.9 μg, respectively. For reverse-phase protein array analysis, at least 10 mg of frozen tumor was required. Standard TCGA criteria included: 1) tumor sample composed predominantly of histologically viable-appearing tumor cells with ≥60% tumor nuclei; 2) ≤20% necrosis of sample volume; and 3) tumor weight ≥100 mg. The Mayo Clinic Institutional Review Board protocol was subsequently modified to reflect the shipment of samples to TCGA’s Biospecimen Core Resource (The Research Institute, Nationwide Children’s Hospital, Columbus, Ohio). Prior to shipping, protected health information was removed from all materials (specimen containers, diagnostic slides, pathology reports). The top slide submitted for review was the diagnostic slide cut from the bisected tumor, representing both the frozen tumor and the FFPE tumor. The initial enrollment, case quality control, other malignancy, and follow-up forms were completed by data coordinators using clinicopathologic variables entered from the REDCap database. Prism (v6.02; GraphPad Software, Inc [commercial software]) was used to generate scatter plots and statistics.

### Analysis of Albumin and Apolipoprotein A-I Oxidation

Albumin S-cysteinylation and apolipoprotein A-I (apoA-I) methionine oxidation were analyzed as described in detail elsewhere [12]. Briefly, samples were thawed, mixed, and then centrifuged to sediment particulates. One-half microliter was removed and diluted 1,000-fold in 0.1% (v/v) trifluoroacetic acid. Five microliters of this solution were injected without delay onto a liquid chromatography—electrospray ionization—mass spectrometry instrument (Eksigent nanoLC*1D connected to a Bruker MicrOTOF-Q operating in time-of-flight only mode) equipped with a protein CapTrap (Optimize Technologies) rather than a conventional column. After flushing small molecules to waste for 3 minutes, albumin and apoA-I were eluted into the mass spectrometer by increasing the acetonitrile concentration over the CapTrap.

Mass spectra containing protein charge envelopes were averaged across approximately 1 minute and then charge-deconvoluted with DataAnalysis (v3.4; Bruker Corp [commercial software]) to a mass range of 1,000 Da on either side of any identified peak. Baseline values were subtracted from deconvoluted spectra and all peak heights were calculated. Tabulated MS peak heights were exported to a spreadsheet for further calculation. The fractional abundance of S-cysteinylated (oxidized) albumin was determined by dividing the height of the MS peak representing S-cysteinylated albumin by the sum of the peak heights for native and S-cysteinylated albumin. ApoA-I methionine oxidation was calculated in an analogous fashion. No MS peaks representing apoA-I with 2 or 3 oxides were detected; however, if they had been, these peaks would have been weighted to calculate total weighted oxidation, as described elsewhere [12].

### ChIP Sequencing

Nephrectomy samples underwent gross macrodissection to ensure that they contained >60% to 70% tumor, with verification by a mirror slide stained with hematoxylin-eosin. Frozen tissues were divided into 50-mg aliquots and stored in 1.5-mL centrifuge tubes at −70°C. Frozen tissues were homogenized on ice for 15–30 seconds in 500 μL 1× phosphate-buffered saline using a tissue grinder (AgileGrinder; ACTGene, Inc). Tissue homogenates were cross-linked using formaldehyde (final concentration, 1%) for 10 minutes; reactions were quenched with 125 mM glycine for 5 minutes at room temperature and washed once with Tris-buffered saline. Pellets were resuspended in cell lysis buffer (10 mM Tris-HCl, pH 7.5; 10 mM NaCl; 0.5% NP-40) and incubated on ice for 10 minutes. Lysates were divided into 2 aliquots and washed with micrococcal nuclease (MNase) digestion buffer (20 mM Tris-HCl, pH 7.5; 15 mM NaCl; 60 mM KCl; 1 mM CaCl_2_). After resuspending in 500 μL MNase digestion buffer containing a proteinase inhibitor cocktail (no. 37491; Active Motif Corp), lysates were incubated in the presence of 1,000 gel units of MNase (no. M0247S; New England Biolabs, Inc) at 37°C for 20 minutes with continuous mixing in a thermal mixer. After adding the same volume of sonication buffer (100 mM Tris-HCl, pH 8.1; 20 mM EDTA; 200 mM NaCl; 2% Triton X-100; 0.2% sodium deoxycholate), lysates were sonicated for 15 minutes (30 seconds on, 30 seconds off) using a Bioruptor Twin (model UCD-400; Diagenode Inc) and centrifuged at 21,130×*g* for 10 minutes. The cleared supernatant (equivalent to 10–20 mg of tissue) was incubated with 2 μg rabbit polyclonal anti—histone H3 lysine 36 trimethylation (H3K36me3) antibody (no. 61101, Active Motif Corp) on a rocker overnight. After adding 30 μL of protein G—agarose beads, reactions were further incubated for 3 hours. Beads were extensively washed with ChIP buffer (50 mM Tris-HCl, pH 8.1; 10 mM EDTA; 100 mM NaCl; 1% Triton X-100; 0.1% sodium deoxycholate), high-salt buffer (50 mM Tris-HCl, pH 8.1; 10 mM EDTA; 500 mM NaCl; 1% Triton X-100; 0.1% sodium deoxycholate), LiCl_2_ buffer (10 mM Tris-HCl, pH 8.0; 0.25 M LiCl_2_; 0.5% NP-40; 0.5% sodium deoxycholate; 1 mM EDTA), and Tris-EDTA buffer. Bound chromatin was eluted and reverse—cross-linked at 65°C overnight. DNA was purified using a MinElute PCR purification kit (no. 28004; Qiagen Inc) after RNase A and proteinase K treatment. H3K36me3 chromatin immunoprecipitation was validated by performing quantitative PCR in the genomic loci targeting the *ACTB* gene body (positive control) and the neighboring intergenic region (negative control). ChIP quantitative PCR was carried out in triplicate on indicated genomic regions using SYBR Green Supermix (Bio-Rad Laboratories, Inc). The following primer sequences were used: hActin: F 5′-CCTCATGGCCTTGTCACAC; hActin: R 5′-GCCCTTTCTCACTGGTTCTCT; hCh19-intergenic: F 5′-AGCTTGTCTTTCCCAAGTTTACTC; hCh19-intergenic: R 5′-TAGCTGTCGCACTTCAGAGGA. The comparative Δ*Ct* method was used to determine relative enrichment compared with input.

ChIP-seq libraries were then prepared from 10 ng ChIP and input DNA using the Ovation Ultralow DR Multiplex kit (NuGEN Technologies Inc). ChIP-seq libraries were sequenced to 51 base pairs (bp) from both ends on an Illumina HiSeq 2000 instrument.

Sequence data were analyzed by the Mayo Clinic Center for Individualized Medicine Bioinformatics Program. The ChIP-Seq pipeline version 2 integrates open-source software packages to analyze ChIP sequencing data and identify profiles from chromatin regulators, posttranslational histone modifications, and transcription factor binding [13]. The source code is publicly available at http://bioinformaticstools.mayo.edu/research/hichipseq-pipeline/, with hyperlinks corresponding to individual software packages. The main features include 1) read-quality checking; 2) read mapping and filtering; 3) library quality assessment; 4) peak calling analysis; and 5) data visualization (S1 Fig). FastQC (http://www.bioinformatics.babraham.ac.uk/projects/fastqc/) (publicly available software) was used to assess the 51-bp paired-end reads for the average quality score per base position. The Burrows-Wheeler Alignment tool (http://bio-bwa.sourceforge.net/) (publicly available software) was used to map the paired-end reads to the reference genome (hg19) [14]. SICER, a package developed for scoring broad binding events (http://home.gwu.edu/~wpeng/Software.htm) (publicly available software), was used to identify H3K36me3 peaks [15]. To plot the genome-wide enrichment, we split the human genome into nonoverlapping 500-bp windows, estimated the number of mapped fragments overlapping each window, and normalized to the number of fragments per 10 million mapped pairs. To profile the level of H3K36me3 occupancy within the gene-body, we downloaded the RefGene annotation from the UCSC Genome Browser table browser (http://genome.ucsc.edu/cgi-bin/hgTables) and intersected that with peak coordinates using BEDTools (http://bedtools.readthedocs.org/en/latest/#) (publicly available) [16].

### RNA Sequencing of Frozen Nephrectomy Specimens

Frozen nephrectomy tissue samples (25–30 mg) were pulverized using a hammer and placed in lysis reagent (Qiazol, no. 79306; Qiagen Inc). RNA from the pulverized tissue was isolated using the miRNeasy Mini Kit (no. 217004; Qiagen Inc) on the QIAcube instrument (no. 9001292; Qiagen Inc). RNA libraries were prepared according to the manufacturer’s instructions for the Encore NGS Library Systems (NuGEN Technologies Inc). Briefly, first-strand cDNA was generated from total RNA using DNA/RNA chimeric primers and reverse transcriptase, creating a cDNA/RNA hybrid. The second-strand cDNA was then synthesized from the DNA/RNA duplex. The resulting double-stranded cDNA was amplified by Single Primer Isothermal Amplification (NuGEN Technologies Inc) using a chimeric primer, DNA polymerase, and RNase H. After amplification, products were modified by random priming and extension to create double-stranded products that were suitable for generating libraries for sequencing. The double-stranded products (200 ng) were fragmented in an ultrasonicator (E210; Covaris Inc) for 5 minutes to generate ~150-bp fragments, which then underwent blunt-end repair. Illumina-compatible paired-end index adapters were ligated to the 5′ and 3′ ends of each fragment. The adapter-modified cDNA fragments were enriched with 5 cycles of PCR amplification. The concentration and size distribution of the resulting libraries were determined by using a Bioanalyzer DNA 1000 chip (Agilent Technologies, Inc). The concentration values were confirmed by Qubit fluorometry (Life Technologies Corp). Libraries were loaded onto Illumina TruSeq v3 paired-end flow cells at concentrations of 8–10 pM to generate cluster densities of 600,000–800,000/mm^2^ following Illumina’s standard protocol and using the Illumina cBot and paired-end cluster kit version 3. Flow cells were sequenced as 100×2 paired-end reads on an Illumina HiSeq 2000 using TruSeq SBS sequencing kit version 3 and HiSeq Control (version 2.0.12.0; Illumina, Inc [commercial software]). Base-calling was performed using Real-Time (version 1.17.21.3; Illumina, Inc [commercial analysis software]).

After proper permissions were obtained, RNA sequencing data on normal and tumor kidney samples were downloaded from TCGA kidney cancer (https://tcga-data.nci.nih.gov/tcga/); Kidney Renal Cell Carcinoma [KIRC] dataset. Sequencing data have been submitted to Gene Expression Omnibus, accession number GSE69198. For meta-analyses, we first normalized reads per kilobase of transcript per million reads mapped (RPKM) values from our frozen-tissue RNA sequencing data, with the standard normalization method of TCGA, ie, RPKM ÷ 75th percentile RPKM value × 1,000.

## Results

### Biobanking Work Flow and Pathologic Characteristics of the RCC Biobank Specimens

**Fig 1:**
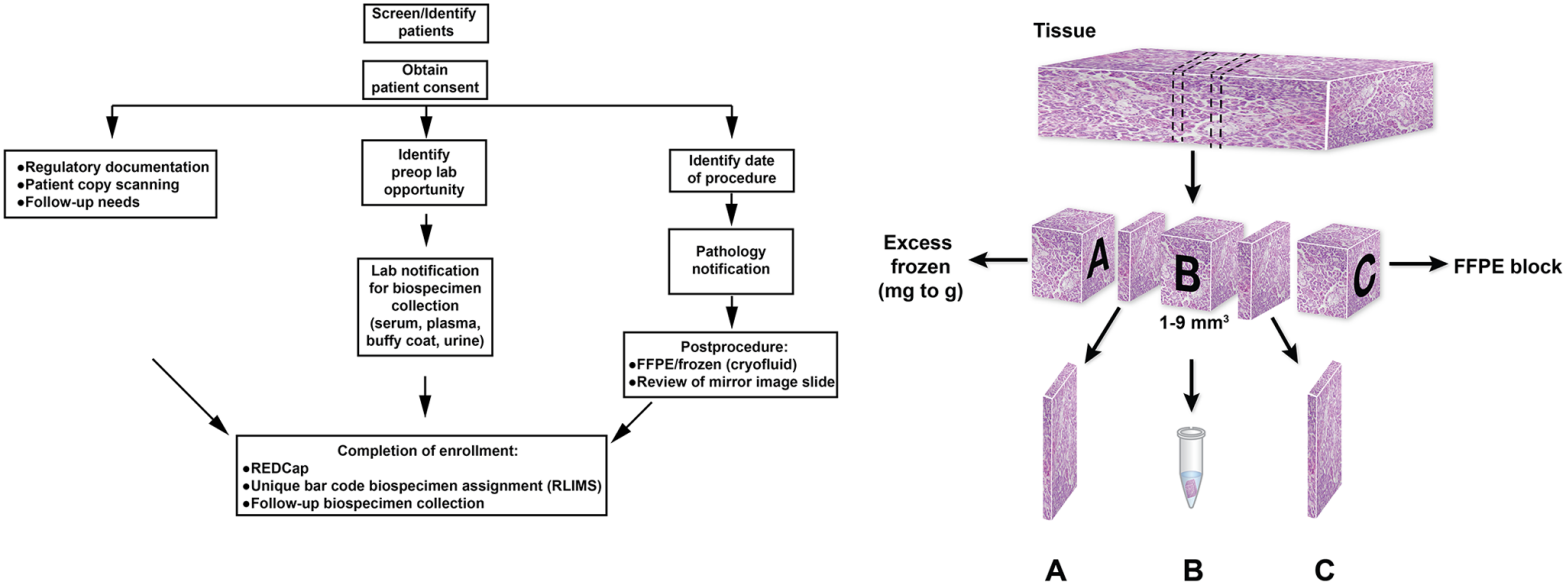
Multidisciplinary Genitourinary Diseases Biospecimen Bank Work Flow. Left, Standard operating procedures involved obtaining patient consent, prospective specimen processing, clinical data collection, and scheduling of future collections associated with standard-of-care blood draws. Right, The pathology work flow for banking frozen tissue. (Data from Pena-Llopis S, Brugarolas J. Simultaneous isolation of high-quality DNA, RNA, miRNA and proteins from tissues for genomic applications. Nat Protoc. 2013 Nov;8[11]:2240–55. Epub 2013 Oct 17.) A portion of the tissue is frozen (A) and the rest is embedded in paraffin (C). A standardized cube of tissue (B) is cut and frozen for future genomic analyses, with parallel mirror slides representing (A) and (C) for assessment of cellularity as an addendum in the pathology synoptic report. Either slide is sent for independent pathology review to the Biospecimen Core Resource of The Cancer Genome Atlas. FFPE, indicates formalin-fixed, paraffin-embedded; lab, laboratory; preop, preoperative; REDCap, Research Electronic Data Capture; RLIMS, Research and Laboratory Information Management System.

Patients undergoing evaluation for genitourinary diseases were recruited and provided consent during urology, radiation oncology, pathology, or medical oncology appointments. Pathologic characteristics of the cohort enrolled from June 1, 2010, to January 1, 2013, are summarized in Table 1. The biobank represents earlier T stages of RCC, consistent with surgical treatments for localized disease. Fig 1 illustrates the work flow for consent of patients, collection and processing of biospecimens, and data input into the centralized REDCap database. To accommodate tissues from diverse anatomical sites, we developed a generalized work flow for collection of frozen tissue, blood, and urine. For tissue, the priority is for diagnostic testing for purposes of routine clinical care, with the rest of the tissue frozen at −80°C in a manner compatible with TCGA tissue collection requirements [17,18]. The protocol is compatible with collection of tissue at the time of surgical resection or biopsy at the primary site (nephrectomy) and at distant metastatic sites.

**Table 1:** Clinicopathologic Characteristics of Renal Cell Carcinoma Specimens Collected in the Multidisciplinary Genitourinary Diseases Biospecimen Bank at Mayo Clinic, Scottsdale, Arizona (N = 328).

Characteristic	Value
Age, median (range), y	66 (32–92)
Sex, No. (%)	
Male	115 (35)
Female	213 (65)
Cancer T stage, No. (%)	
T1a	176 (54)
T1b	67 (20)
T2a	15 (5)
T2b	7 (2)
T3a	40 (12)
T3b	13 (4)
T3c	3 (1)
T4	7 (2)
Pathology, No. (%)	
Clear cell	216 (66)
Papillary	36 (11)
Oncocytoma	36 (11)
Chromophobe	20 (6)
Angiomyolipoma	13 (4)
Other^a^	7 (2)

^a^ Includes unclassified, mucinous, and tubular spindle cell carcinoma.

### TCGA Specimen Submission and Quality Control

**Fig 2:**
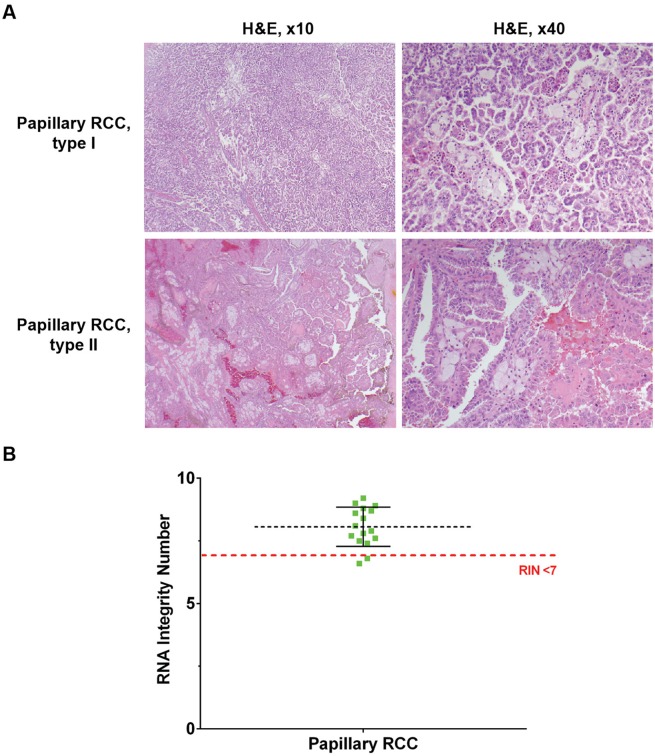
Papillary Renal Cell Carcinoma (RCC) Samples Submitted to The Cancer Genome Atlas (TCGA). A, Representative mirror images of top slides sent for independent pathology review to the Biospecimen Core Resource of TCGA. H&E indicates hematoxylin-eosin stain; original magnification ×10 (left) and ×40 (right). B, Scatterplot of TCGA quality assessment of RNA processed from papillary RCC specimens. The black horizontal dotted line represents the mean, with the vertical error bars representing the SD. RNA integrity numbers (RINs) were assessed by TCGA RNA bioanalyzer. Two samples with RIN <7 were disqualified.

Frozen papillary RCC samples were submitted for processing to TCGA. As a control, buffy coat collected concomitant with tissue was submitted as a germline control. The genitourinary pathologist reviewed the diagnostic slide, which is a mirror image of the frozen tissue and FFPE, to ensure that samples met tumor-specific pathology requirements. The mean (SD) tumor weight was 376 (130) mg. DNA and RNA yields from tumor and matched buffy coat are listed in Table 2. Representative diagnostic slides were sent to TCGA (Fig 2). All extracted DNA samples passed the TCGA requirements. With our work flow, 2 of 16 submitted tumors (12.5%) did not meet RNA integrity number (RIN) requirements (RIN<7). Our mean (SD) TCGA RINs were 8.1 (0.8) for papillary RCC. Table 3 lists the genomic platforms used to analyze TCGA samples with publicly accessible data.

**Table 2:** DNA and RNA Yields From Samples Submitted to The Cancer Genome Atlas.

Sample ^a^	Yield, mean (SD), μg
Tumor DNA	28.6 (14.9)
Buffy coat DNA	48.0 (22.4)
Tumor RNA	46.9 (10.3)

^a^ The mean (SD) tumor weight was 376 (130) mg.

**Table 3:** Papillary Renal Cell Carcinoma Samples With Available Genomic Data from The Cancer Genome Atlas Data Portal^a^.

TCGA ID	Clinical Information	DNA Methylation	SNP Array	MicroRNA Sequencing	RPPA	RNA Sequencing
TCGA-SX-A7SL	✓	✓	✓	✓		✓
TCGA-SX-A7SM	✓	✓	✓	✓	✓	✓
TCGA-SX-A7SN	✓	✓	✓	✓	✓	✓
TCGA-SX-A7SO	✓	✓	✓	✓	✓	✓
TCGA-SX-A7SP	✓	✓	✓	✓	✓	✓
TCGA-SX-A7SQ	✓	✓	✓	✓	✓	✓
TCGA-SX-A7SR	✓		✓			
TCGA-SX-A7SS	✓	✓	✓	✓	✓	✓
TCGA-SX-A7SU	✓	✓	✓	✓		✓
TCGA-SX-A71R	✓	✓	✓	✓	✓	✓
TCGA-SX-A71S	✓	✓	✓	✓		✓
TCGA-SX-A71U	✓	✓	✓	✓	✓	✓
TCGA-SX-A71V	✓	✓	✓	✓		✓
TCGA-SX-A71W	✓		✓			

Abbreviations: ID, identifier; RPPA, reverse phase protein array; SNP, single nucleotide polymorphism; TCGA, The Cancer Genome Atlas.

^a^ Entries with checkmarks indicate availability as of 4/22/15 from TCGA Data Portal.

### Analysis of Albumin and apoA-I Oxidation as a Metric of Plasma Specimen Integrity

**Fig 3:**
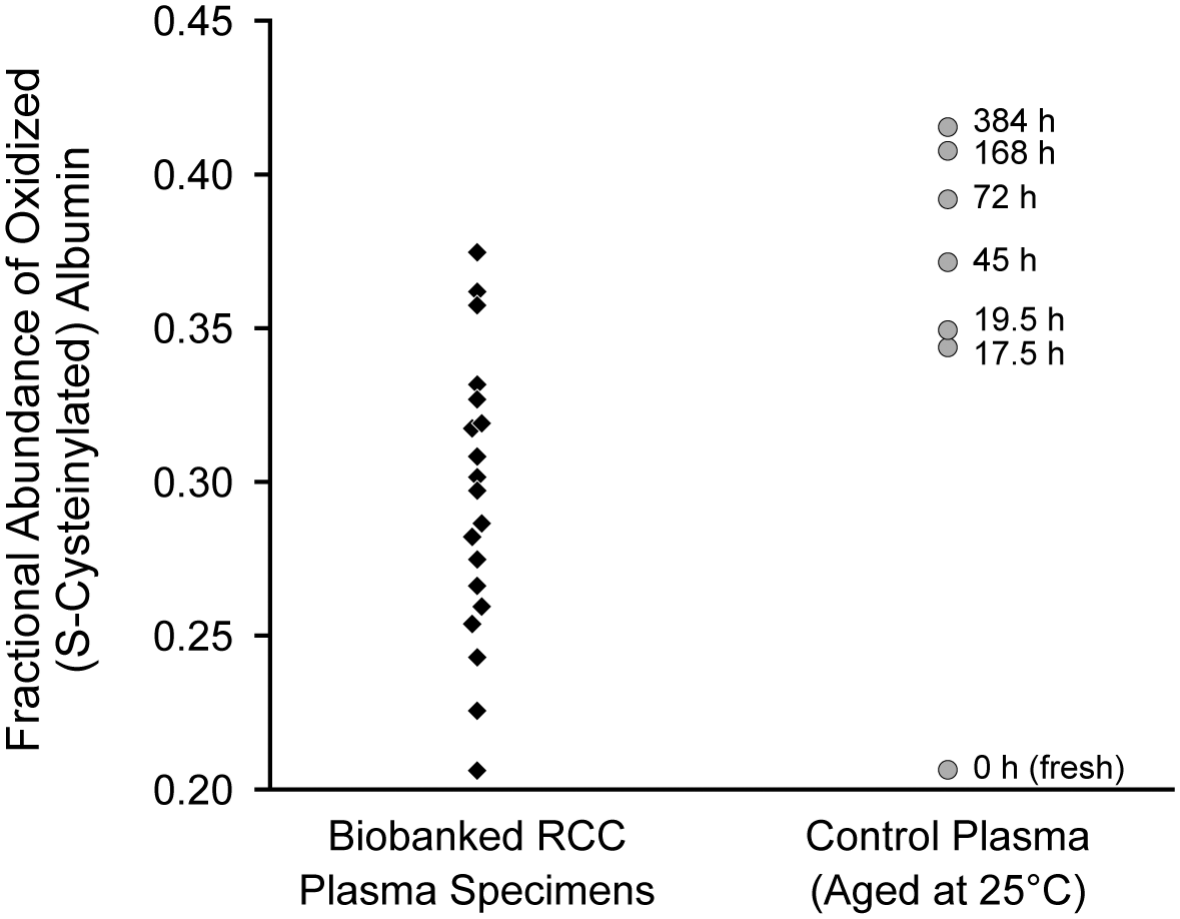
Albumin Oxidation (S-Cysteinylation) Observed in the Banked Renal Cell Carcinoma (RCC) Plasma Samples. Control plasma was freshly collected from a nominally healthy donor, and albumin oxidation levels were observed at 25°C Significantly less albumin oxidation was observed in samples from patients with RCC compared with albumin in plasma kept at 25°C for ≥17.5 hours (*P*<.001; Mann-Whitney U test). (Data from Borges CR et al [12].)

As previously reported [12], albumin and apoA-I in human blood plasma are susceptible to oxidative S-cysteinylation and methionine sulfoxidation, respectively, when plasma is exposed to temperatures above its freezing point of −30°C. At both room temperature and −20°C, albumin oxidation (S-cysteinylation) increases as per the complement of an exponential decay function before reaching a plateau (for most samples) at a relative fractional abundance of about 0.4. ApoA-I methionine oxidation proceeds at a slower pace, requiring days of exposure to room temperature or months of storage at −20°C [12]. On average, the banked samples from patients with RCC described in this study had albumin oxidation (S-cysteinylation) levels consistent with several hours’ exposure to room temperature and no long-term storage above −30°C (Fig 3). Consistent with this observation, apoA-I oxidation remained below quantifiable levels for tested samples.

### H3K36me3 Profiling by ChIP Sequencing

**Fig 4:**
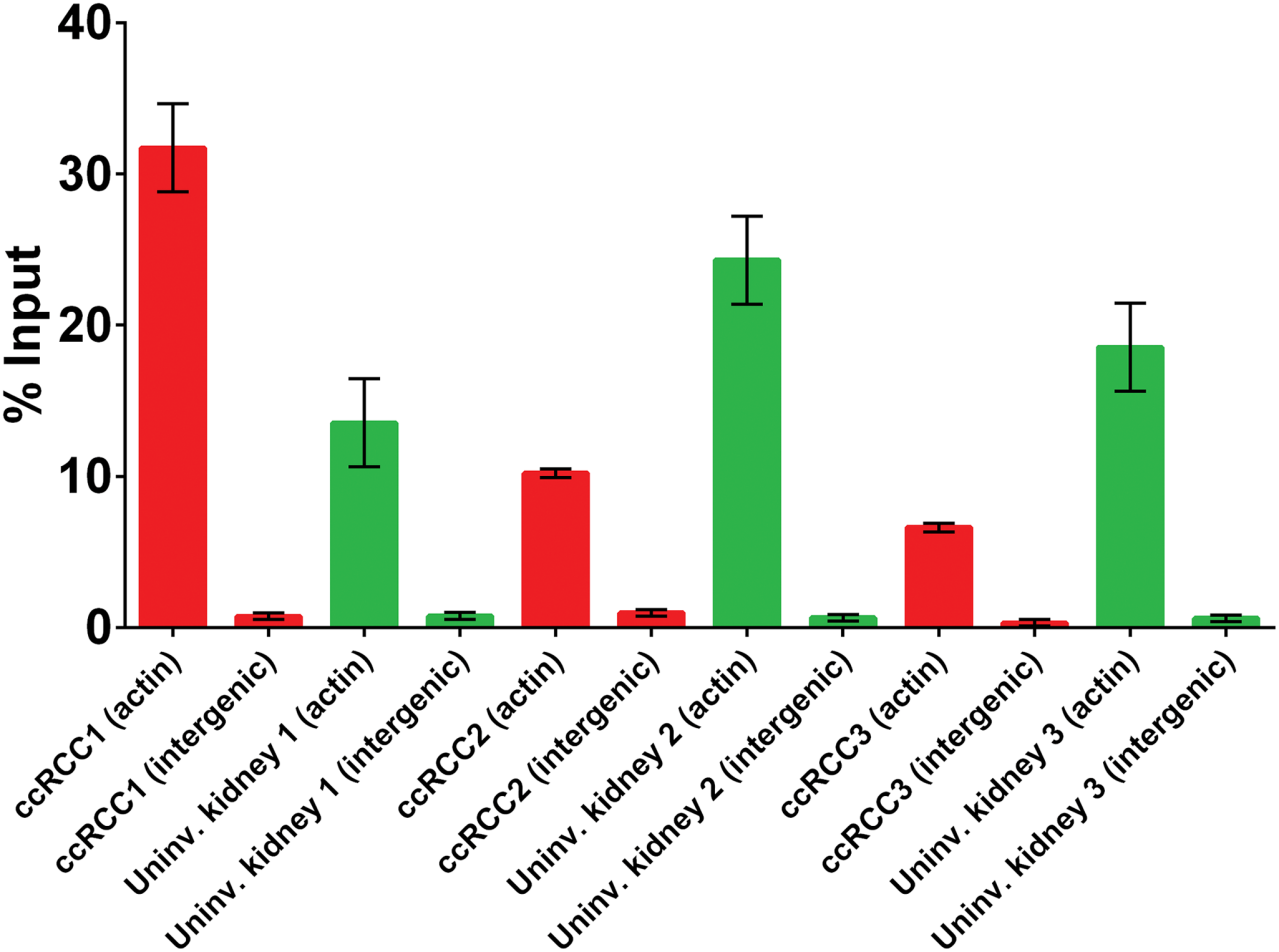
Chromatin Immunoprecipitation Quantitative Polymerase Chain Reaction Analysis of Histone H3 Lysine 36 Trimethylation (H3K36me3). H3K36me3 is enriched at the actin locus, but not in the intergenic region, in both tumor and uninvolved kidney. Error bars represent SD as determined from duplicate experiments. ccRCC indicates clear cell renal cell carcinoma.

We tested the utility of our banked specimens for emerging technologies such as ChIP sequencing that require larger amounts of frozen tissue (>100 mg). Altered chromatin accessibility is observed in clear cell RCC and is associated with alterations of H3K36me3 [19–22]. Compared to other genome-wide discovery platforms, ChIP sequencing often requires larger amounts of tissue (>100 mg). To determine whether our biobanking work flow for specimens was compatible with H3K36me3 ChIP sequencing, we performed ChIP quantitative polymerase chain reaction to ensure quality and yield of immunoprecipitated chromatin from 6 samples. An H3K36me3 antibody was used for immunoprecipitation of endogenous H3K36me3-bound DNA. Since H3K36me3 is enriched at exons and active genes, polymerase chain reaction primers were optimized for active genes, such as actin, and for an intergenic region (negative control) [23,24]. At a minimum, 4-fold enrichment is required before proceeding to sequencing [25,26]. We found that actin was enriched 8- to 32-fold over the intergenic controls (Fig 4), which indicated that the quality and yield of immunoprecipitated DNA/chromatin complexes were sufficient for generating ChIP-sequencing libraries. S2 Fig shows the optimal 100–400 bp DNA fragments that were compatible with ChIP sequencing.

**Fig 5:**
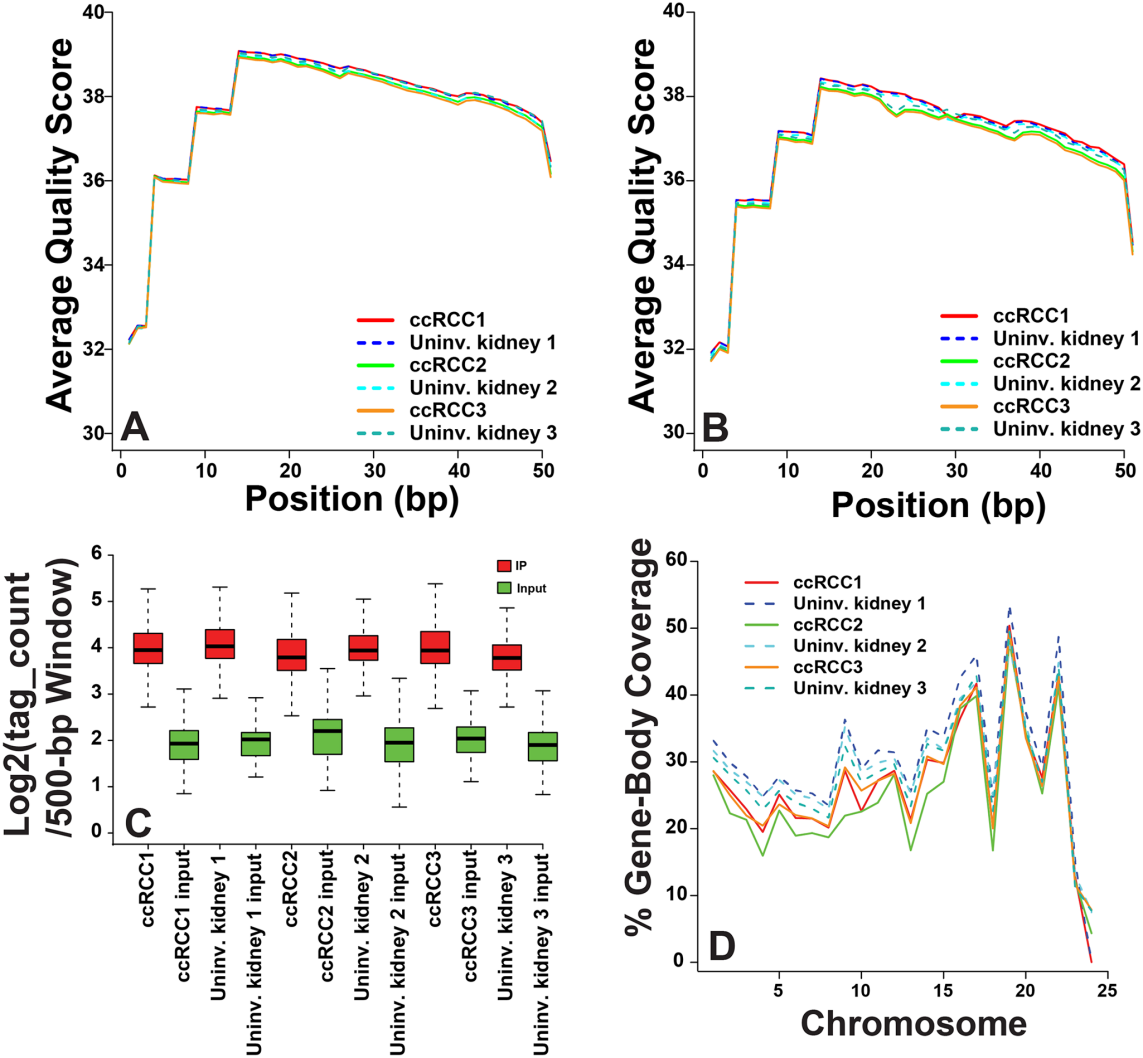
Quality Assessment of Chromatin Immunoprecipitation Coupled With High-throughput Sequkencing. A, First end-read and, B, second end-read average quality score per base pair (bp) position assessment using FastQC. Higher scores correspond to better base calls. C, Box plots of enrichment of H3K36me3 immunoprecipitation (IP; red) over the matched control input library (Input; green). The human genome was split into 500-bp nonoverlapping windows, and the number of mapped pairs per window was calculated using BEDTools and normalized to a library size of 10 million uniquely mapped reads. The plots represent the top 5% of the 500-bp windows with the highe st counts in IP and the corresponding windows in input. D, Gene-body coverage by H3K36me3-binding sites. H3K36me3-binding sites identified by SICER (Spatial Clustering for Identification of ChIP-Enriched Regions) were intersected with gene coordinates to calculate the gene-body coverage (y-axis). On the x-axis, 1 to 22 represents chromosomes 1 to 22; 23 represents the X chromosome; and 24 represents the Y chromosome. ccRCC1 indicates clear cell renal cell carcinoma 1; ccRCC2, clear cell renal cell carcinoma 2; ccRCC3, clear cell renal cell carcinoma 3; Uninv., uninvolved.

To evaluate the properties of ChIP-sequencing libraries derived from banked frozen nephrectomy samples, we checked paired-end read quality, genome-wide enrichment level, and distribution of H3K36me3 occupancy. Both reads had good quality, with FastQC scores of 32 to 39 across all 51 bp (Fig 5A and 5B). The first 3 bp had the lowest quality score for both reads, with the first read having slightly better sequencing quality, as is typically seen for paired-end sequencing. In addition, over 95% of the first reads had an average quality score of ≥30 compared to 91% to 92% of the second reads. Only a small proportion of the reads had poor quality scores (≤20, on average): <0.5% for the first read and <3% for the second, respectively (data not shown). Each library had 27.0 to 30.6 million pairs of raw reads, and 91.7% to 92.8% of the pairs mapped to the reference genome (hg19) by BWA. Of these, 85.4% to 86.7% represented uniquely mapped pairs.

For ChIP-sequencing experiments, another quality measurement is the level of enrichment between immunoprecipitated DNA and the input DNA control. The box plots represent the top 5% of the 500-bp windows with the highest number of fragments (Fig 5C). The peak regions from each H3K36me3 immunoprecipitated library showed an average of about 4-fold enrichment over the corresponding regions in the input library. In published ChIP-sequencing studies in cell lines, H3K36me3 is predominantly associated with gene bodies [23,27]. To map H3K36me3 in gene bodies, we examined the distribution of H3K36me3 across each chromosome. The 3 RCC libraries showed a 2% to 5% reduction of H3K36me3 occupancy over the gene body, compared to matched normal samples (Fig 5D). H3K36me3 is enriched at exons, and the difference between different chromosomes in H3K36me3 occupancy over the gene body largely reflected the ratio of exon sizes over gene sizes. For example, chromosome 19 had the highest size ratio of exon/gene (13.6%) and also the highest H3K36me3 coverage of gene body (47.4%-53.5%). Similar patterns were also found for chromosomes 16 and 22.

### RNA Sequencing Quality of Frozen Nephrectomy Samples

To assess the quality of RNA sequencing data from frozen nephrectomy samples, we analyzed RNA from frozen tissue profiled in parallel by ChIP sequencing (N = 6). A representative bioanalyzer profile and RIN of isolated RNA from paired samples (tumor and normal) is depicted in S3 Fig. We first examined the sequencing quality per base for forward and reverse reads. As shown in S4 Fig, the average Phred score of each base in all reads was above 30, indicating a high-quality sequencing run. Next, we compared the quality of RNA sequencing from our frozen tissues with that of an RCC cell line (786-O), which was prepared under optimal RNA isolation conditions. After genome alignments, only 0.2%-0.4% of total reads from frozen tissues failed the default quality check steps of Bowtie2 (disqualified reads are shown in Table 4), which was comparable to the disqualification ratio (0.1%) of the 786-O cell line. The mapping ratios for our frozen tissues were slightly lower (76.4%-83.8%) than those from the cell line (84.5%).

**Table 4:** Comparison of Mapped Reads Between Frozen Nephrectomy Samples and a Renal Cell Carcinoma Cell Line (786-O).

Source and Sample ID	Total Reads	Disqualified Reads		Unmapped Reads		Mapped Reads	
		Count	% Total	Count	% Total	Count	% Total
Kidney tumor							
ccRCC1	414,293,024	1,801,533	0.4	79,723,359	19.2	332,768,132	80.3
Uninv kidney 1	421,101,208	1,018,419	0.2	68,354,681	16.2	351,728,108	83.5
ccRCC2	427,575,250	1,535,886	0.4	99,270,525	23.2	326,768,839	76.4
Uninv kidney 2	426,317,444	1,205,535	0.3	67,952,300	15.9	357,159,609	83.8
ccRCC3	409,282,398	1,565,821	0.4	92,300,799	22.6	315,415,778	77.1
Uninv kidney 3	424,633,572	878,592	0.2	75,578,922	17.8	348,176,058	82.0
Cell line							
786-O	164,760,980	149,964	0.1	25,449,107	15.4	139,161,909	84.5

Abbreviations: ccRCC1, clear cell renal cell carcinoma 1; ccRCC2, clear cell renal cell carcinoma 2; ccRCC3, clear cell renal cell carcinoma 3; Uninv, uninvolved.

**Fig 6:**
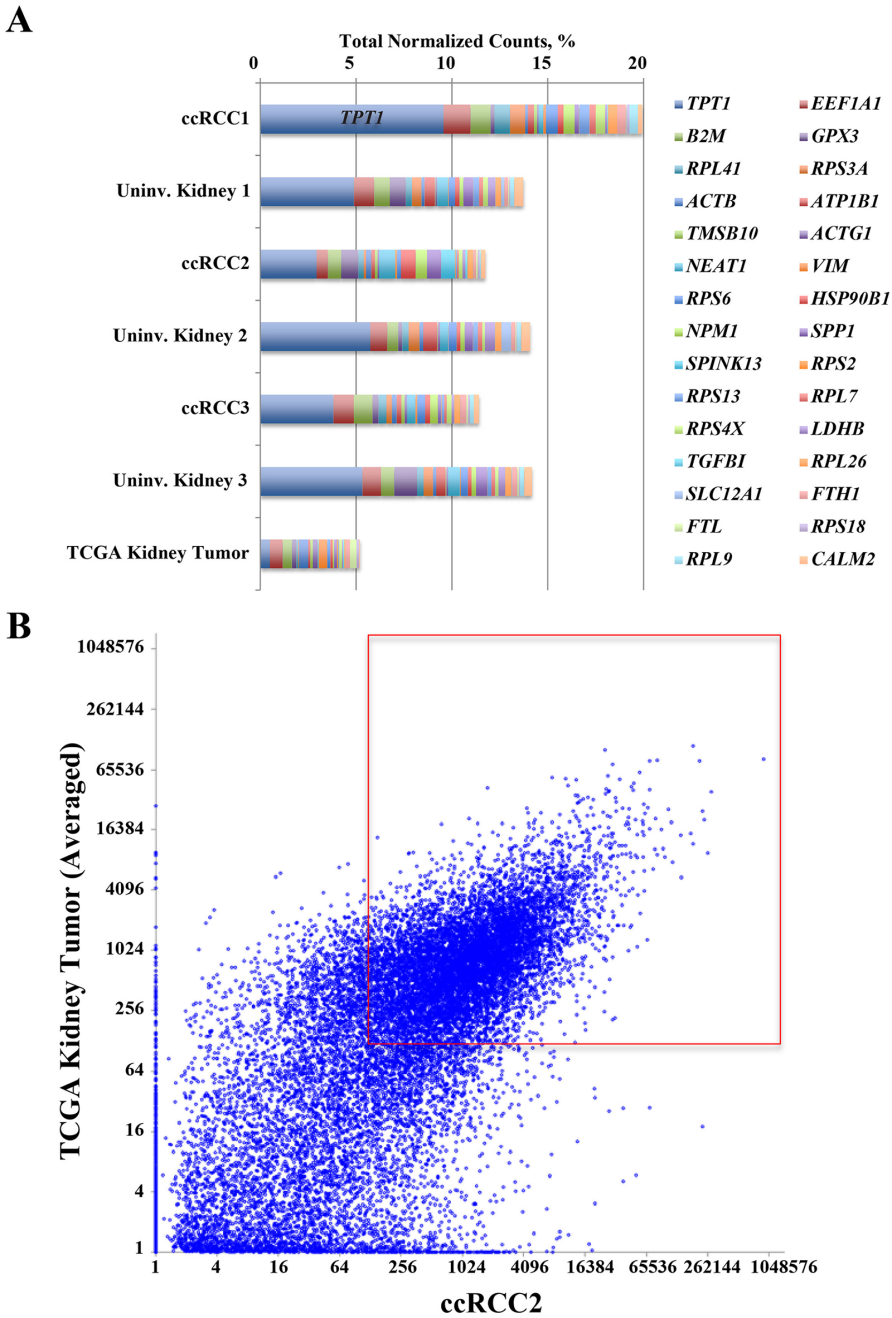
Expression Profiles and Correlation of Genes in the Mayo Clinic and TCGA Cohorts. A, Expression of the most abundant genes in Mayo Clinic tissues. Expression levels of the 30 most-abundant genes in frozen tumors (upper 6 bars) and TCGA kidney tumors (averaged from 553 samples) are shown in stacked bars, with percentages of total normalized counts. Reads for *TPT1* occupied up to 9.5% of total reads. B, Correlation of gene expression between the Mayo Clinic and TCGA kidney tumors. Normalized expression values of a representative Mayo Clinic tumor and TCGA kidney tumor samples (averaged from 553 samples) were compared. Genes in the boxed area have normalized expression values ≥128. ccRCC1 indicates clear cell renal cell carcinoma 1; ccRCC2, clear cell renal cell carcinoma 2; ccRCC3, clear cell renal cell carcinoma 3; TCGA, The Cancer Genome Atlas; Uninv, uninvolved.

One of the key metrics of RNA sequencing quality and coverage is the number of identified genes. To identify genes expressed in both the Mayo Clinic and TCGA cohorts with high confidence, we counted genes with normalized expression values ≥128 that corresponded approximately to an average of 1.0 RPKM across all samples. As shown in Table 5, among ~20,500 protein-coding genes, we observed a similar number of expressed genes in the Mayo Clinic and TCGA tissues. Poor-quality RNA sequencing often results in biased amplification and consequent overrepresentation of a subset of genes. To assess the RNA sequencing quality, expression levels of the most abundant genes across all samples were examined. Fig 6A shows that *TPT1*, *EEF1A1*, *B2M*, and *GPX3* were most abundant in both the Mayo Clinic and TCGA samples. With the exception of *TPT1* (highly abundant in the Mayo Clinic samples and accounted for up to 9.5% of expression values of total normalized counts), we did not identify any other overrepresented genes. Despite fundamental differences in sample source, sample processing, and sequencing library generation, the overall correlation coefficients between the Mayo Clinic vs TCGA tissues ranged from 0.75 to 0.82 (Fig 6B). Although expression levels of low-abundance genes were slightly overestimated in the Mayo Clinic samples, genes with mid to high abundance were more linearly aligned. These data support the compatibility of our biobanking protocol with RNA and ChIP-sequencing platforms for frozen RCC.

**Table 5:** Comparison of Mapped Reads Between the Mayo Clinic and TCGA Nephrectomy Samples.

Sample Group	No. of Samples	No. of Genes With Normalized Score ≥128, Mean (SD)
Mayo kidney tumor	6	12,177.0 (317.2)
TCGA kidney tumor	533	11,339.8 (324.8)
TCGA normal kidney	72	11,444.5 (172.9)

Abbreviation: TCGA, The Cancer Genome Atlas.

## Discussion

The Multidisciplinary Genitourinary Diseases Biospecimen Bank in Arizona is a collaboration among investigators in urology, radiation oncology, pathology, and medical oncology to study the spectrum of benign and malignant genitourinary disease. Our work flow suggests that our biobanking protocol for RCC does not interfere with routine clinical care and is compatible with systematic cancer genomics projects such as TCGA and MS analysis of plasma, as well as with newer technologies (eg, ChIP sequencing) requiring larger quantities of frozen tissue. Herein, we reported the quality control of our TCGA submissions for papillary RCC (paired frozen tissue, mirror slide, and buffy coat), MS analysis of albumin S-cysteinylation as a surrogate of plasma integrity, and ChIP sequencing analysis of frozen tissues.

With matched temporal collections of frozen tissue and buffy coat, we can link molecular characterizations of DNA, RNA, and protein to treatment outcomes. The annotation of tumor cellularity of matched FFPE specimens in the pathology report expedites the identification of tumor blocks suitable for targeted exome sequencing, and immunohistochemistry [28–30]. Integration of the clinical information into a REDCap database facilitates collaborations to query specific disease cohorts linked to outcomes data.

### Limitations

Our study has several limitations. First, our study represents biospecimens processed within 4 years of collection. The biobank started prospective collections in 2010, and our DNA and RNA yields, MS analysis, and ChIP sequencing may not be representative of samples stored longer than 4 years or of other genitourinary tumors (eg, urothelial, testicular, penile, or prostate cancers). For ChIP sequencing, we chose a covalent histone modification, H3K36me3, for enrichment of DNA; our H3K36me3 ChIP enrichment may not be comparable to ChIP enrichment using antibodies directed to transcription factors, which may not be as stable in banked frozen tissue. Our RNA yields reflect an overall mean warm-ischemia time of 45.8 minutes (range, 10–110 minutes) for the RCC specimens (N = 328), which was influenced by factors such as partial vs radical nephrectomy or an open vs a minimally invasive approach. Despite the warm-ischemia time, the overall rate of sample disqualification for RIN <7 for TCGA submissions was 12.5%. In a prior study, RNA degradation in nephrectomy specimens was observed primarily after 4 hours at 37°C, and prolonged warm ischemia was associated with changes in gene expression profiles [31]. Furthermore, we studied only nephrectomy specimens; other tissues may have different sensitivities to warm-ischemia times.

Second, we examined the compatibility only of frozen nephrectomy specimens, rather than archival FFPE nephrectomy specimens, with various discovery platforms. The use of FFPE has additional challenges that include fragmentation and degradation of nucleic acids, and alterations of epitopes during the fixation and deparaffinization process. FFPE-compatible DNA, RNA, and ChIP sequencing protocols for various tissues have been published, but further study is required to determine the compatibility of these protocols with FFPE nephrectomy tissue [32–34].

Third, elevated concentrations of circulating free cysteine and cystine (the disulfide dimer of free cysteine) have been reported in patients with severely compromised renal function [35]. Their effects on albumin S-cysteinylation in vivo are not entirely clear because the ratio of cysteine to cystine in blood does not vary much [36], presumably because the redox potential of blood plasma is rather tightly regulated. Ex vivo, however, elevated concentrations of cysteine and cystine in the presence of oxygen would almost certainly lead to higher-than-normal maximum levels of S-cysteinylated albumin through disulfide exchange with cystine and through sulfenic acid—mediated disulfide bond formation with cysteine [37–40]. In some patients with renal failure, the fractional abundance of S-cysteinylated albumin has been reported at over 50%, although these studies lacked detailed documentation of sample-handling conditions [41–44]. Essentially, this means that elevated ex vivo levels of albumin S-cysteinylation are likely to occur faster in patients with renal failure than in patients without elevated plasma cysteine or cystine.

## Conclusion

Genitourinary diseases represent a spectrum of disease with varying phenotypes and genotypes. The increase in tumor molecular profiling has led to a need for integration of genotype-phenotype relationships with contemporary treatments (definitive surgical management, medical therapy, and/or radiation). The construction of a biobank that encompasses both benign and malignant genitourinary diseases may also elucidate the mechanism of the molecular progression of disease prior to overt clinical symptoms. Our multidisciplinary collaboration ensures longitudinal collection of matched tissue, blood, and urine during the course of standard care as well as during experimental treatments that will be available for projects that require access to pre- and/or postintervention time points. The prospective collection of frozen biospecimens allows future next-generation platforms to interrogate specimens linked to outcomes. A comprehensive prospective collection also facilitates banking of frozen tumors with rare subtypes and histologies, which may not be identified until completion of dedicated immunohistochemical staining (ie, not intraoperatively). Furthermore, the comparison of benign and malignant tissue may yield clues regarding risk-based algorithms for personalized treatment. The elucidation of distinct molecular phenotypes of RCC will further improve upon the prognostic and therapeutic stratification of patients.

## Supporting Information

S1 Fig Flowchart of Mayo Clinic ChIP-Seq pipeline. The integrative analysis uses datasets from ChIP libraries and a reference genome. The main features include 1) read-quality checking; 2) read mapping and filtering; 3) library quality assessment; 4) peak calling analysis; and 5) data visualization. The source code is available at http://bioinformaticstools.mayo.edu/research/hichipseq-pipeline/. ChIP indicates chromatin immunoprecipitation; H3K36me3, histone H3 lysine 36 trimethylation.
(DOC)

S2 Fig DNA Fragment Analysis of Nephrectomy ChIP Libraries and Input Chromatin. Before library preparation, the majority of input chromatin is from mononucleosomes with a DNA fragment size of 100–200 bp. After library preparation for ChIP sequencing, the target fragment size is 100–400 bp. bp indicates base pair; ccRCC1, clear cell renal cell carcinoma 1; ccRCC2, clear cell renal cell carcinoma 2; ccRCC3, clear cell renal cell carcinoma 3; ChIP, chromatin immunoprecipitation; H3K36me3, histone H3 lysine 36 trimethylation; Uninv, uninvolved.
(DOC)

S3 Fig Bioanalyzer Data of RNA Isolated From Nephrectomy Tissue. A, ccRCC1 with RIN = 8.6. B, Uninvolved kidney with RIN = 8.6. The 28s/18s rRNA ratios are given, with gel images to the right. ccRCC1 indicates clear cell renal cell carcinoma 1; FU, fluorescence unit; RIN, RNA integrity number.
(DOC)

S4 Fig Sequencing Quality Scores per Base. Phred scores per base for forward (A) and reverse (B) reads of a representative sample are shown. Red dotted lines represent the threshold (score = 30) for good-quality sequencing. Plots were generated by FASTQC software. Solid red lines indicate median values; solid blue lines, mean values; boxes, 25th to 75th percentiles.
(DOC)

S1 File Consent Form for the Research Study. (DOC)
