# Supplementary figures and images for: A Multidisciplinary Biospecimen Bank of Renal Cell Carcinomas Compatible with Discovery Platforms at Mayo Clinic, Scottsdale, Arizona
Source: PLoS One. 2015 Jul 16;10(7):e0132831. doi: 10.1371/journal.pone.0132831 (PMC4504486; doi:10.1371/journal.pone.0132831)

**
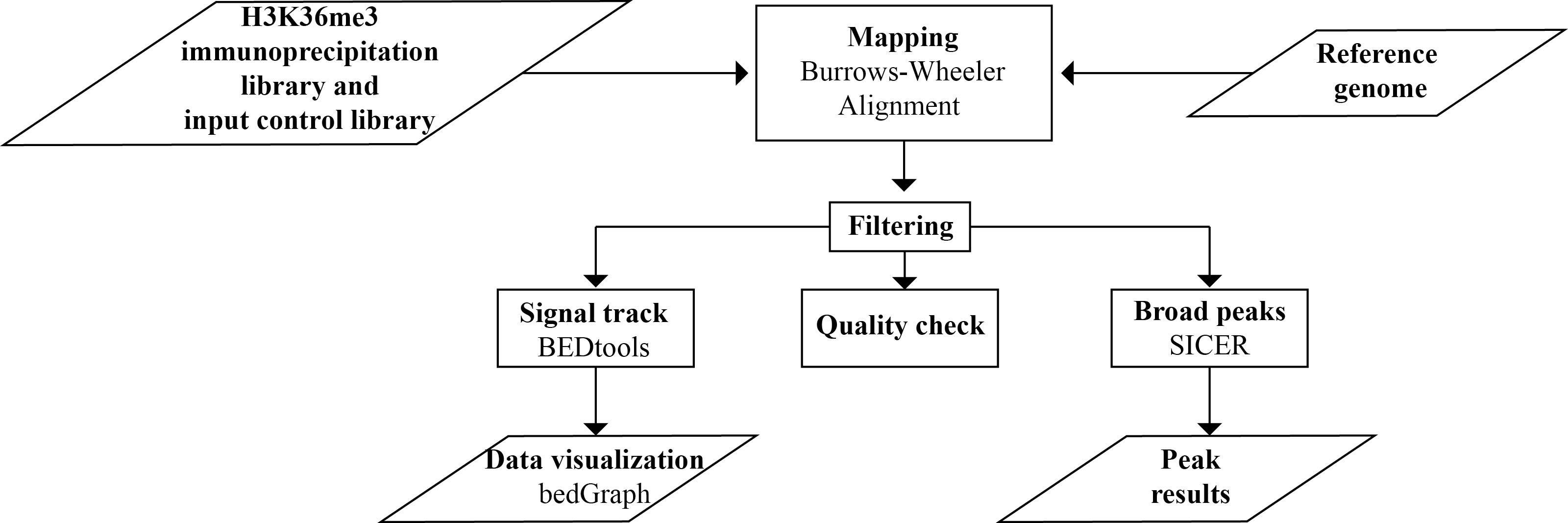
**

Supplement: S1 Fig — The integrative analysis uses datasets from ChIP libraries and a reference genome. The main features include 1) read-quality checking; 2) read mapping and filtering; 3) library quality assessment; 4) peak calling analysis; and 5) data visualization. The source code is available at http://bioinformaticstools.mayo.edu/research/hichipseq-pipeline/. ChIP indicates chromatin immunoprecipitation; H3K36me3, histone H3 lysine 36 trimethylation. (DOC) [file pone.0132831.s001.doc]

**
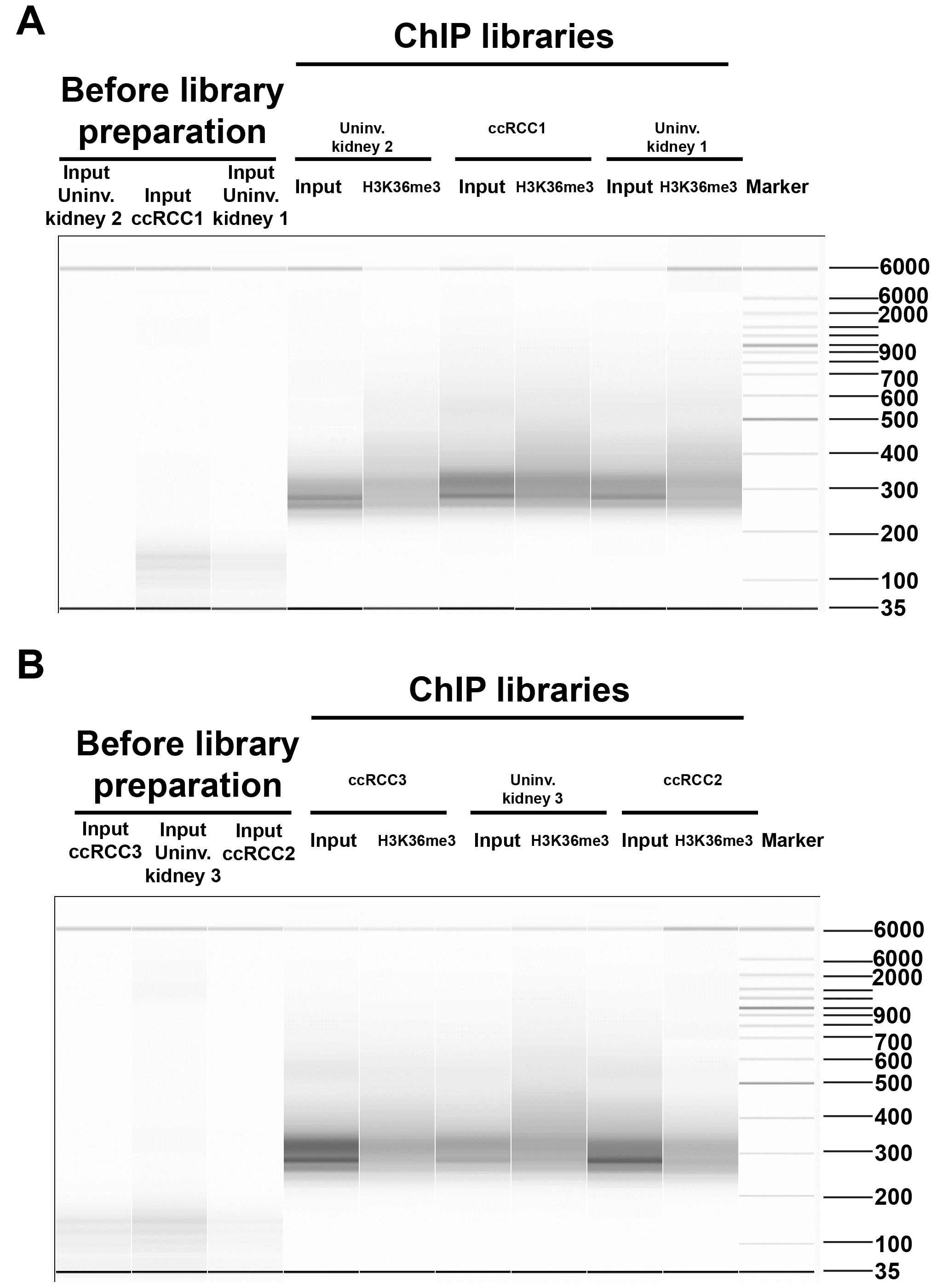
**

Supplement: S2 Fig — Before library preparation, the majority of input chromatin is from mononucleosomes with a DNA fragment size of 100–200 bp. After library preparation for ChIP sequencing, the target fragment size is 100–400 bp. bp indicates base pair; ccRCC1, clear cell renal cell carcinoma 1; ccRCC2, clear cell renal cell carcinoma 2; ccRCC3, clear cell renal cell carcinoma 3; ChIP, chromatin immunoprecipitation; H3K36me3, histone H3 lysine 36 trimethylation; Uninv, uninvolved. (DOC) [file pone.0132831.s002.doc]

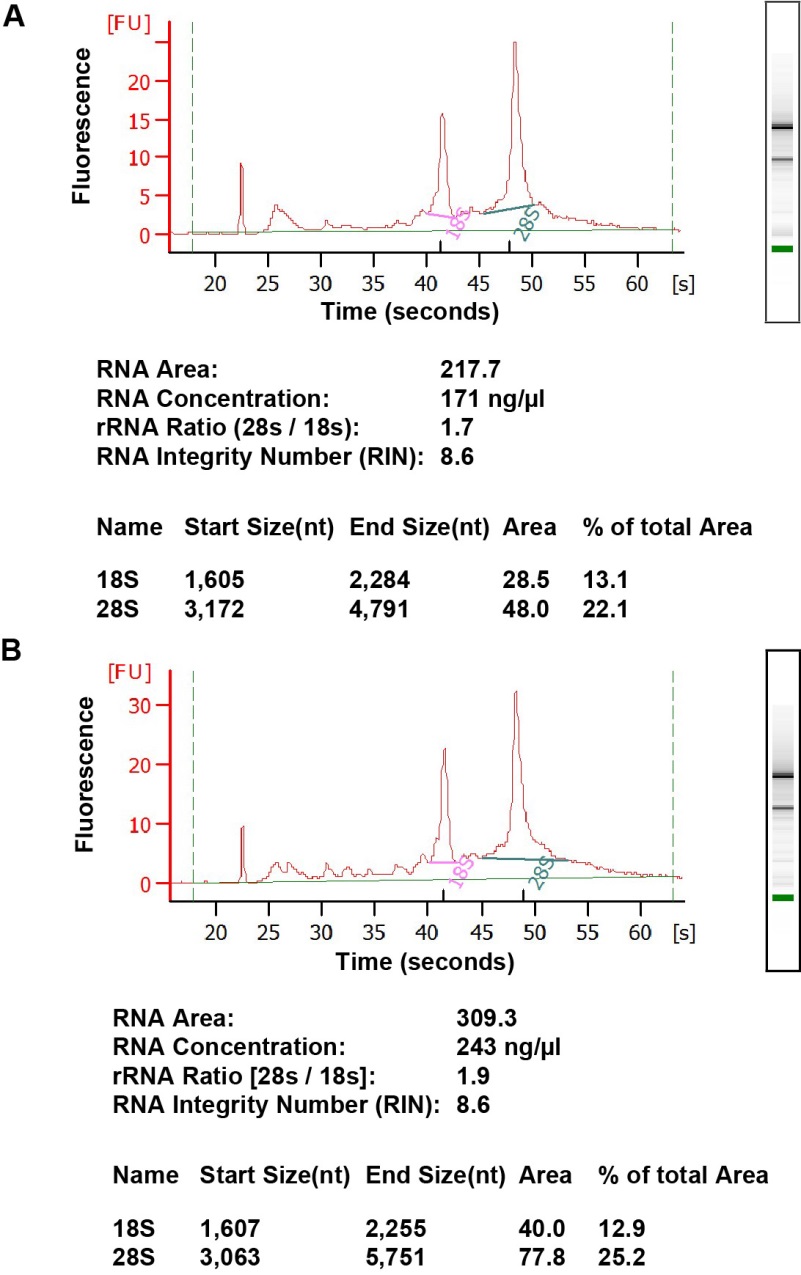

Supplement: S3 Fig — A, ccRCC1 with RIN = 8.6. B, Uninvolved kidney with RIN = 8.6. The 28s/18s rRNA ratios are given, with gel images to the right. ccRCC1 indicates clear cell renal cell carcinoma 1; FU, fluorescence unit; RIN, RNA integrity number. (DOC) [file pone.0132831.s003.doc]

**
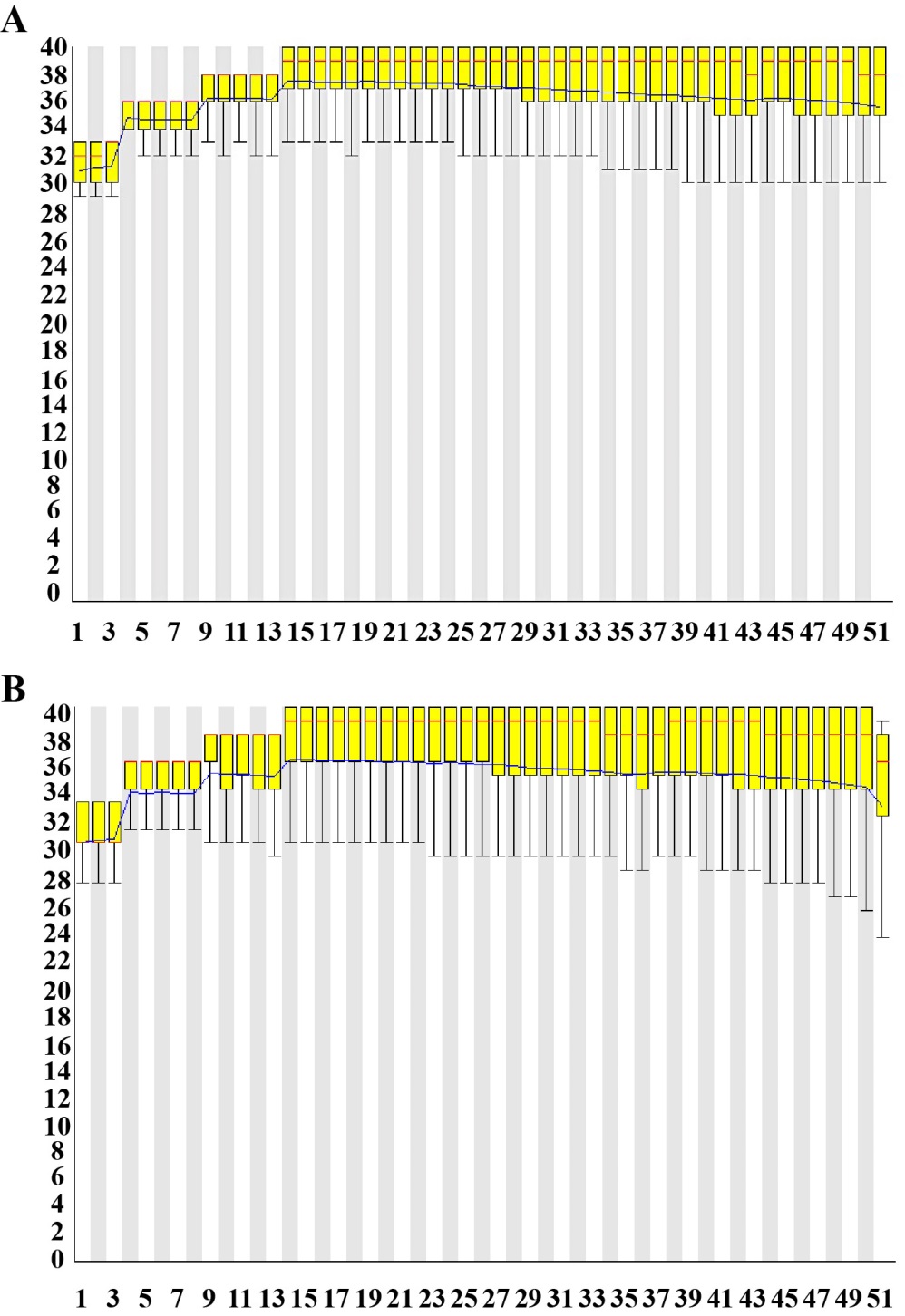
**

Supplement: S4 Fig — Phred scores per base for forward (A) and reverse (B) reads of a representative sample are shown. Red dotted lines represent the threshold (score = 30) for good-quality sequencing. Plots were generated by FASTQC software. Solid red lines indicate median values; solid blue lines, mean values; boxes, 25th to 75th percentiles. (DOC) [file pone.0132831.s004.doc]
