# Supplementary material for: A Multidisciplinary Biospecimen Bank of Renal Cell Carcinomas Compatible with Discovery Platforms at Mayo Clinic, Scottsdale, Arizona
Source: PLoS One. 2015 Jul 16;10(7):e0132831. doi: 10.1371/journal.pone.0132831 (PMC4504486; doi:10.1371/journal.pone.0132831)
Supplement: S1 File — (DOC) [file pone.0132831.s005.doc]

Name and Clinic Number

IRB No. **08-000980-00***

Consent form approved **December 14, 2012**.

This consent valid through **December 13, 2013**.

**1. General Information About This Research Study**

**Study Title:** Benign and Malignant Genitourinary Diseases Biospecimen Bank

**Name of Principal Investigator on This Study:** Dr. Erik P. Castle and Colleagues

**A. Study Eligibility and Purpose**

You are being asked to take part in this research study because you may have a genitourinary disease. Genitourinary disease is a disease of the urinary system or reproductive organs.

As you read this form describing the study, ask any questions you have. Take your time to decide. Feel free to discuss the study with your family, friends, and health care provider before you decide. You may stop participating at any time during the study. You may decide not to participate. If so, none of your current benefits or normal health care will be affected in any way. When you feel comfortable that all your questions have been answered, and you wish to take part in this study, sign this form in order to begin your participation. If you are agreeing for someone else, you need to sign this form. Your signature means you have been told about the study and what the risks are. Your signature on this form also means that you want yourself, or your child/relative/principal/ward to take part in this study.

* This document is copyrighted by Mayo Foundation for Medical Education and Research. Used with permission.

**B. Number of Participants**

The plan is to have as many people as possible to take part in this study at Mayo Clinic.

**2. What Will Happen To You While You Are In This Research Study?**

If you agree to be in the study, you will be asked to participate in the following:

If you are to have a surgery/surgeries as part of your routine clinical care, excess tissue and/or excess kidney stones remaining after the clinical pathology has been reviewed may be stored as part of this study for future research at Mayo Clinic, Scottsdale, Arizona. Tissue will be collected only if you are scheduled for a routine surgical procedure. You will not have tissue collected solely for research purposes.

If you are to have a routine clinical care blood sample obtained from a vein in your arm, at the same time, 30 mL (approximately 2 tablespoons) will be withdrawn and stored for future research at Mayo Clinic, Scottsdale, Arizona. You will not have blood collected solely for research purposes.

If you are to have a routine clinical care urine sample collected, any remaining urine may be stored as part of this study for future research at Mayo Clinic, Scottsdale, Arizona. You may have urine samples collected solely for research purposes.

If you require future surgery, routine blood, and/or urine samples needed for the diagnosis, treatment, and follow-up for the routine clinical care of your disease, we will collect similar research samples at that time as well.

Information from your medical record will be collected and stored in a deindentified, password-protected electronic database.

**3. How Long Will You Be In This Research Study?**

You will be in the study for/until as long as it takes to collect your tissue, blood, and urine samples.

**4. Why You Might Want To Take Part In This Research Study**

This study will not make your health better. It is for the benefit of research.

**5. What Are The Risks Of This Research Study?**

Your doctor will discuss the risks of surgery, blood, and urine sample collections, as these tests and procedures are part of your standard clinical care.

**A. Pregnancy and Birth Control**

1) Will women of child-bearing potential be allowed to participate in this study?

**Yes**: Women of child-bearing potential will be able to participate in this study.

2) Will pregnant and/or nursing women be allowed to participate in this study?

**Yes**: Women who are pregnant and/or nursing may take part in this study.

3) Do you need to have a pregnancy test done to be part of the study?

**No**: The risk to an unborn child does not exist. Pregnant women are eligible to take part in this study.

4) Will men who are able to father a child be allowed to participate in this study?

**Yes**: Men who are able to father a child are allowed to take part in this study.

**B. Risk Summary**

There are no known physical risks to you from taking part in this research study. There is a slight risk of loss of privacy/confidentiality, but measures will be taken to prevent this from happening. Specimens will be stored with a code (personal information, such as your name, will be removed), and all information will be stored in a password-protected database.

**6. What Other Choices Do You Have If You Don’t Take Part In This Research Study?**

This study is only being done to gather information. You may choose not to take part in this study.

**7. Are There Reasons You Might Leave This Research Study Early?**

Taking part in this research study is your decision. You may decide to stop at any time. You should tell the researcher if you decide to stop and you will be advised whether any additional tests may need to be done for your safety.

In addition, the researchers or Mayo Clinic may stop you from taking part in this study at any time:

- If it is in your best interest.
- If you do not follow the study rules.
- If the study is stopped.

**8. Will You Need To Pay For Any Of The Tests And Procedures?**

You will not need to pay for tests and procedures that are done just for this research study. These tests and procedures are:

- - Urine samples collected solely for research purposes
  - Storage of your samples

**9. Will You Be Paid For Participating In This Research Study?**

You will not be paid for taking part in this study.

**10. What Happens If You Are Injured Or Ill Because You Were In This Research Study?**

If you have side effects from taking part in this study, you need to report them to the researcher and your regular physician, and you will be treated as needed. Mayo will give medical services for treatment for any bad side effects from taking part in this study. Such services will be free if not covered by a health plan or insurance. No additional money will be offered.

No side effects are expected from taking part in this study. Your participation only involves the donation of your samples.

**11. What Are Your Rights If You Are In This Research Study?**

Taking part in this research study will not change your rights and benefits. Taking part in this research study does not give you any special privileges. If you decide to not participate in this study, or stop in the middle of the study, no benefits will be taken away from you. Specifically, you do not have to be in this research study to receive or continue to receive medical care from Mayo Clinic.

You will be told of important new findings or any changes in the study or procedures that may affect you or your willingness to continue in the study.

**12. What About Your Privacy?**

# A. Authorization To Use And Disclose Protected Health Information

Your privacy is important to us, and we want to protect it as much as possible. By signing this form, you authorize Mayo Clinic and the investigators to use and disclose any information created or collected in the course of your participation in this research protocol. This information might be in different places, including your original medical record, but we will only disclose information that is related to this research protocol for the purposes listed below.

This information will be given out for the proper monitoring of the study, checking the accuracy of study data, analyzing the study data, and other purposes necessary for the proper conduct and reporting of this study. If some of the information is reported in published medical journals or scientific discussions, it will be done in a way that does not directly identify you.

This information may be given to other researchers in this study, or to private, state, or federal government parties or regulatory authorities in the USA and other countries responsible for overseeing this research. These may include the US Food and Drug Administration, the Office for Human Research Protections or other offices within the US Department of Health and Human Services, and the Mayo Clinic Office for Human Research Protection or other Mayo Clinic groups involved in protecting research subjects.

If this information is given out to anyone outside of Mayo Clinic, the information may no longer be protected by federal privacy regulations and may be given out by the person or entity that receives the information. However, Mayo Clinic will take steps to help other parties understand the need to keep this information confidential.

This authorization lasts forever unless you rescind (stop) it.

You may stop this authorization at any time by writing to the following address:

Mayo Clinic

Office for Human Research Protection

ATTN: Notice of Revocation of Authorization

200 1st Street SW

Rochester, MN 55905

If you stop authorization, Mayo Clinic may continue to use your information already collected as part of this study, but will not collect any new information.

**13. What Will Happen To Your Samples?**

Your samples of tissue, blood, and/or urine will be kept at Mayo Clinic for use in this study. Researchers at Mayo Clinic who are not involved with this study may ask to use your samples for more research. You have a say in how your stored samples are used in future research.

**A. Identification Information**

If you agree to allow your samples to be used for further research, the samples may be stored forever. The samples will be stored at Mayo Clinic and would be given a code (instead of your name) while they are stored and when they are used in research. This code allows your samples to be used without anyone knowing that they are your samples just by looking at the label.

There is a very small chance that some commercial value may result from the use of your donated samples. If that happens, you will not be offered a share in any profits.

**B. Risks**

Some future studies may be for testing the genes you inherited from your parents (also known as genetic testing). If a researcher finds that future test results may be useful for your health care, you will be contacted and given the choice to learn the test results. At that time, you will be given general information on the potential risks, benefits, and costs of choosing to learn the test results. The risks of learning genetic test results may include emotional upset, insurance or job discrimination, and/or family conflicts from learning unknown information about your parents or blood relatives. Test results will only be put into your medical record if you choose to learn the results. Sometimes results should be released only through a genetic counselor, who can help explain the possible risks and benefits of learning the results.

**C. Exceptions (when your samples may be used without your permission)**

1) When government rules allow your samples to be used without identifying you, even with a code.

2) When use of the samples is not considered human subject research.

At all other times:

- You can let Mayo Clinic use your samples.
- You can say NO to having your samples used by Mayo Clinic.

**D. Please read the following statements and mark your choice:**

1) I permit my samples to be stored and used in future research of genitourinary diseases at Mayo Clinic:

Yes  No Please initial here: ________Date: ________

2) I permit my samples to be stored and used in future research at Mayo Clinic to learn about, prevent, or treat any other health problems:

Yes  No Please initial here: ________Date: ________

**E. Who will use your samples?**

If you agree to give your samples, they will become the property of Mayo Clinic and may be used for research by Dr Erik P. Castle and other staff at Mayo Clinic. Researchers at other institutions may also ask for a part of your samples for future studies.

**F. How do researchers from other institutions get the samples?**

Researchers from universities, hospitals, and other health organizations conduct research using tissue. They may contact Mayo Clinic and request samples for their studies. If you approve release of your samples by checking “yes” below, Mayo Clinic may send the tissue samples and some information about you to researchers who request them, but Mayo Clinic will not send your name, address, phone number, Social Security number, or any other identifying information with the sample. If you allow your samples to be given to researchers at other institutions, they will be given with a code number rather than your name. If these researchers use the samples for future research and decide that a test result may be useful for your health care, they may contact Mayo Clinic and Mayo Clinic would then contact you to offer you the choice to learn the test results. Mayo Clinic has the right to end storage of these samples without telling you.

I permit Mayo Clinic to give my samples to researchers at other institutions:

***Please mark one box:***

Yes  No Please initial here: ________Date: ________

**G. If you want your samples destroyed at any time, write to:**

Dr Erik P. Castle

Urology Department, Mayo Clinic

MCSB

5777 East Mayo Boulevard

Phoenix, AZ 85054

**H. If you move, please send your new address to:**

Mayo Clinic

Section of Registration

13400 East Shea Boulevard

Scottsdale, AZ 85259

**14. Who Can Answer Your Questions?**

| **You can call:** | **At:** | **If you have questions or concerns about:** |
| --- | --- | --- |
| Principal Investigator:  Dr Erik P. Castle | Phone:  (480) 342-2805 | The study tests and procedures  Research-related injuries or emergencies  Any research-related concerns or complaints |
| Mayo Clinic IRB  Research Subject Advocate | Phone:  (507) 266-4000  Toll-Free:  (866) 273-4681 | Rights of a research subject  Use of protected health information  Any research-related concerns or complaints |
| Research Billing | Arizona:  (800) 603-0558 | Billing/Insurance |

**15. Summary and Enrollment Signatures**

You have been asked to take part in a research study at Mayo Clinic. The information about this study has been provided to you to inform you about this study.

- I have read the whole consent form, and all of my questions have been answered to my satisfaction.
- I am satisfied that I have been given enough information about the purpose, methods, risks, and possible benefits of the study to decide if I want to join.
- I know that joining the study is voluntary and I agree to join the study.
- I know that I can call the investigator and research staff at any time with any questions or to tell them about side effects.
- I know that I may withdraw from the study at any time.
- A copy of this form will be put in my medical records.
- I will be given a copy of this completed form.

Please sign and date to show that you have read all of the above guidelines. Please do not sign unless you have read this entire consent form. If you do not want to sign, you don’t have to; but if you don’t, you cannot participate in this research study.

______________ __________________________________________ ________________

(Date/Time) (Printed Name of Participant) (Clinic Number)

_____________________________________________

(Signature of Participant)

______________ _______________________________________________

(Date/Time) (Printed Name of Individual Obtaining or in Receipt of Consent)

_______________________________________________

(Signature of Individual Obtaining or in Receipt of Consent)
